# The Impact of Obesity on the Excretion of Steroid Metabolites in Boys and Girls: A Comparison with Normal-Weight Children

**DOI:** 10.3390/nu15071734

**Published:** 2023-04-01

**Authors:** Marta Sumińska, Rafał Podgórski, Piotr Fichna, Artur Mazur, Marta Fichna

**Affiliations:** 1 Department of Pediatric Diabetes, Auxology and Obesity, Institute of Pediatrics, Poznan University of Medical Sciences, 60-572 Poznan, Poland; 2 Doctoral School, Poznan University of Medical Sciences, 60-812 Poznan, Poland; 3 Centre for Innovative Research in Medical and Natural Sciences, University of Rzeszow, 35-310 Rzeszow, Poland; 4 Department of Biochemistry, Institute of Medical Sciences, College of Medical Sciences, University of Rzeszow, 35-959 Rzeszow, Poland; 5 Department of Pediatrics, Childhood Endocrinology and Diabetes, Collegium of Medical Sciences, University of Rzeszow, 35-959 Rzeszow, Poland; 6 Department of Endocrinology, Metabolism and Internal Medicine, Poznan University of Medical Sciences, 60-355 Poznan, Poland

**Keywords:** obesity, nutrition, urinary steroid metabolites, biomarkers, childhood, adolescence, boys, girls, puberty

## Abstract

Obesity in childhood is associated with several steroid changes, which result from excess body mass. The aim of this study was to evaluate steroid metabolism in children with obesity compared with those with normal weight, especially in relation to sex and puberty progress. We analyzed the clinical data of 191 children, aged between 5 and 18 years, with 115 affected (64 girls and 51 boys) and 76 unaffected (35 girls and 41 boys) by obesity. Routine clinical assessment and pubertal stage evaluation based upon Tanner’s scale were performed. In addition, to evaluate the impact of puberty, children with pre-adolescence and advanced puberty were divided into separate subgroups. Then, 24 h urine steroid excretion profiles were analyzed by gas chromatography/mass spectrometry. Significant differences in the excretion of steroid metabolites were found between normal weight children and children with obesity, especially in the prepubertal cohort. In this group, we observed enhanced activity in all the pathways of adrenal steroidogenesis. Raised excretion of mineralocorticoid derivatives such as tetrahydro-11-deoxycorticosterone, tetrahydrocorticosterone, and 5α-tetrahydrocorticosterone supported increased activity of this track. No significant differences were detected in the excreted free forms of cortisol and cortisone, while the excretion of their characteristic tetrahydro-derivatives was different. In pre-adolescent children with obesity, α-cortol and especially α-cortolone appeared to be excreted more abundantly than β-cortol or β-cortolone. Furthermore, in children with obesity, we observed elevated androgen excretion with an enhanced backdoor pathway. As puberty progressed, remarkable reduction in the differences between adolescents with and without obesity was demonstrated.

## 1. Introduction

The increasing prevalence of childhood obesity, which was further exacerbated by lockdowns during the COVID-19 pandemic, remains a major challenge for the healthcare system [1,2,3]. Obesity is a pathological process characterized by multifactorial etiology. Subsequently, it can contribute to multifaceted metabolic disorders and chronic health problems [3,4,5]. The tendency for binge eating is often noted in people with a high BMI, which may be accompanied by alteration in the signaling of appetite-regulating hormones [6]. Such disturbances may be also induced by glucocorticoid overexposure, for example, due to chronic stress, disease, or medication use [7,8]. Obesity, which develops already in childhood, seems to be associated with several steroid changes, which may both contribute to and be the result of excess body mass [9].

Steroid hormones affect the entire body and are involved in different biological pathways, mainly in the proper function as well as in metabolic homeostasis maintenance. Their regulation is complex and is based on even opposing phenomena, including hormone production and degradation, rate of activation vs. inactivation, and the ratio between free and bound circulating steroid compounds [10,11]. Many studies have proved that obesity is associated with abnormalities in the hypothalamic–pituitary–adrenal (HPA) axis, including enhanced susceptibility to its activation [12,13,14]. In a systematic review, Incollingo Rodriguez et al. suggested that HPA axis activity and obesity are interrelated; however, the literature for this subject is highly inconsistent [15]. On the other hand, Tenk et al. demonstrated that excess body mass does not lead to exacerbated HPA axis activity [16]. The relationship between alterations in diurnal cortisol rhythm and obesity appears to be better understood, although there are still conflicting reports. Some data show an increase in cortisol levels in obesity [17,18,19,20], while others indicate no association or even inverse correlations [21,22,23,24]. As a matter of fact, despite many years of research focused on steroid biology, the knowledge about their synthesis, metabolism, and excretion remains full of gaps.

A highly important point is that adipose tissue is currently considered as a biologically active endocrine organ synthesizing hormones, growth factors, and numerous peptides called adipokines, which act within the adipose tissue (autocrine and paracrine) as well as in distant organs and tissues (classical endocrine action). Leptin and adiponectin are best known for their metabolic action. Low leptin levels elicit a physiological starvation response including feelings of hunger, reduced energy expenditure, and increased enjoyment of food. Adiponectin’s role in appetite regulation is not fully elucidated, as studies indicate both anorexigenic and orexigenic effects [8]. Several studies provide evidence that adipokines may affect adrenal steroid secretion or impact their metabolism in the liver [25,26,27,28].

**Figure 1:**
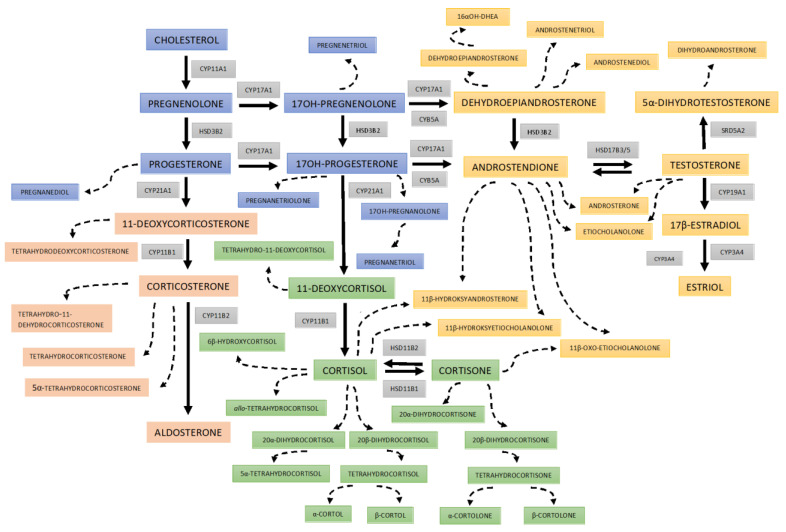
Scheme of 24 h urine steroid excretion. The blue boxes bear the names of the steroid precursors at the early stages of steroidogenesis; reddish rectangles for the mineralocorticosteroid pathway and its metabolites; green boxes for cortisol and its metabolites; and yellowish areas are background for androgens and their precursors. The broken lines indicate the urine metabolites of the steroid hormones and precursors. The abbreviations of the enzymes involved in each step are displayed in gray boxes: CYP11A1—cytochrome P450 cholesterol monooxygenase (side-chain-cleaving); HSD3B2—3β-hydroxysteroid dehydrogenase type 2; CYP21A2—cytochrome P450 21α-hydroxylase; CYP11B1—cytochrome P450 11β-hydroxylase type 1; CYP11B2—cytochrome P450 11β-hydroxylase type 2 as part of aldosterone synthase; CYP17A1—cytochrome P450 17α-hydroxylase/17,20-lyase; HSD11B1—11β-hydroxysteroid dehydrogenase type 1 (mainly reductase 11BHSD); HSD11B2—11β-hydroxysteroid dehydrogenase type 2 (mainly oxidase 11BHSD); CYB5A cytochrome b5; HSD17B3/5—17β-hydroxysteroid dehydrogenase type 3/type 5; SRD5A2—steroid 5α-reductase type 2; and CYP19A1—cytochrome P450 aromatase; CYP3A4—cytochrome P450 16α-hydroxylase.

The biosynthesis, metabolism, and excretion of steroid hormones are very complex (Figure 1). Cortisol concentrations can be assessed in plasma, serum, urine, saliva, and even hair [17,18,19,20,21,22,23,24,29,30,31]. However, it seems that 24 h urine collection is the most reliable method for comprehensive evaluation of various steroid metabolites, even in the era of fast liquid chromatography tandem mass spectrometry [32,33]. Gas chromatography-mass spectrometry (GC-MS) has excellent resolution and is routinely used to analyze steroid metabolite profiles in urine, especially in pediatrics, where it is highly valued for the diagnosis of rare steroid disorders with high diagnostic sensitivity and specificity [31,33]. Steroid urine profiling provides a longer overview, not just point insight, and allows for a more complete analysis of multiple metabolites simultaneously. Furthermore, the urinary profile can be investigated without the burden of stress effects, which may affect the results, especially in children. Although individual steroids have been routinely investigated for the diagnosis of endocrine disorders for many years, recent technological advances allow simultaneous determination of numerous metabolites. This approach may better reflect their origin and metabolic balance, hence it is fundamental for the more appropriate interpretation of the obtained data, as well as for precise diagnosis [34].

Our study was aimed at analyzing the steroid urinary excretion of metabolites in a group of children suffering from obesity and those with normal body mass, who were further stratified according to sex and pubertal stage. Previous studies exploring this topic featured smaller patient cohorts with no distinction of their age, and fewer analyzed steroid metabolites [35,36].

## 2. Research Design and Methods

The study comprised 191 children aged between 5 and 18 years (Table 1). The group with obesity consisted of 115 patients (64 girls and 51 boys) hospitalized in the Department of Pediatric Diabetes, Auxology and Obesity in the University Children’s Hospital. Obesity was defined as BMI values above the 97th percentile of the BMI reference channels developed for the population of Polish children and adolescents. Patients with obesity secondary to endocrine disorders or genetic syndromes, as well as those under current medications or special diets, were excluded from the study. All patients underwent a routine clinical assessment including general physical examination, basic anthropometric measurements, and evaluation of the pubertal stage based upon the Tanner’s scale. The examination was followed by 24 h urine collection according to the standard protocol, performed at home to avoid the extra stress connected with hospitalization. Children and parents were primed in the collection procedure and received appropriate written instructions to ensure compliance.

The control group comprised 76 children (35 girls and 41 boys) with normal weight, defined as BMI values between the 15th and 85th percentiles of the same reference channels for Polish children and adolescents. Individuals were recruited from children of the hospital employees and their families, who were capable of giving attention to the 24 h urine collections. Children with any chronic disease or special diets as well as those under current medications were not recruited.

In addition, to assess the impact of puberty, children with pre-adolescence (Tanner 1) and advanced puberty (Tanner 3, 4, and 5) were selected from the entire study group. Individuals at Tanner stage 2 were excluded from this analysis to avoid interference of the intermediate results. The prepubertal group consisted of 60 children (24 girls and 36 boys), including 24 with normal body weight and 36 with obesity. For the group with advanced puberty we assigned 95 children (55 girls and 40 boys), including 27 with normal and 68 with excessive body mass.

The study was conducted in line with the Declaration of Helsinki and approved by the Bioethical Committee at Poznan University of Medical Sciences (No 866/20, 10 December 2020). Informed consent was obtained from the legal representatives and from the participants aged at least 16 years.

### 2.1. Quantification of the Urinary Steroid Metabolites

Steroid metabolites in the 24 h urine samples were analyzed using targeted GC-MS, as previously described [37]. In brief, free and conjugated urinary steroids were extracted by solid phase extraction, and then conjugates were enzymatically hydrolyzed with sulfatase and β-glucuronidase/arylsulfatase. After the addition of known amounts of internal standards (stigmasterol and cholesteryl butyrate), methoxyamine-trimethylsilyl ether derivatives were formed. Medroxyprogesterone was used as a recovery standard. GC-MS was performed with a Shimadzu QP-2010 Ultra Plus gas chromatograph. The analytes were separated through a ZB-1ms column and detected in a selected ion monitoring mode.

### 2.2. Statistical Analyses

Clinical data and 24 h urinary excretion of steroid metabolites in each group were analyzed using Statistica 13.3 (StatSoft Inc., Tulsa, OK, USA). Data were tested for normality of distribution, and the Mann–Whitney U test was applied to determine differences between groups. The level of statistical significance was accepted as *p*-value < 0.05. The most appropriate classifier for distinguishing steroid metabolic disorders in children with obesity and healthy volunteers was assessed based on the area under the curve (AUC) and its 95% confidence interval (CI) in receiver operating characteristic (ROC) analysis using MedCalc software version 20.218 (Ostend, Belgium). The box plots were also created in MedCalc software.

## 3. Results

The clinical characteristics of normal weight controls and participants with obesity are shown in Table 1. No statistically significant difference in mean age was found between individuals with and without obesity. BMI, Z-score BMI, and body surface area (BSA) were significantly higher in the group of patients with obesity than in normal weight children.

Thirty-seven steroid metabolites were compared between cohorts (Table 2). Sex-stratified analyses were also performed (Table 3 and Table 4). Furthermore, the prepubertal boys and girls and those with advanced puberty were evaluated separately (Supplementary Tables S1–S4).

**Figure 2:**
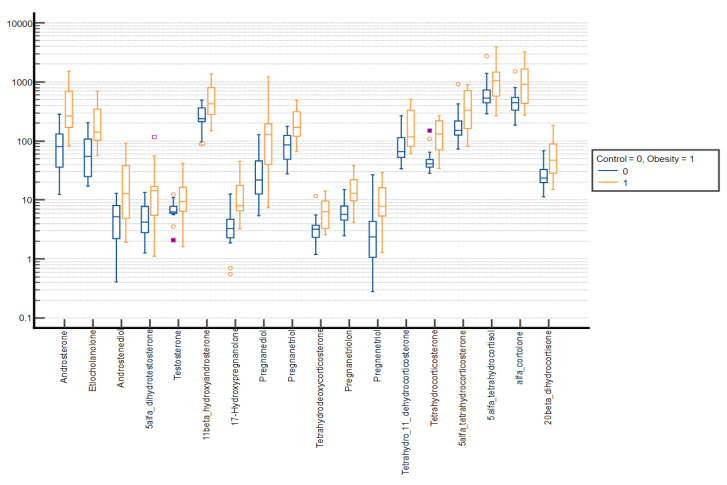
Comparative box plots of steroid metabolites with statistically significant differences (*p*-value < 0.05) between the normal weight and obesity boys in the prepubertal group. A logarithmic scale was used. Exact results are available in Supplementary Table S1. Yellow circle—outliers; purple square—far outliers; hollow box- extreme values.

**Figure 3:**
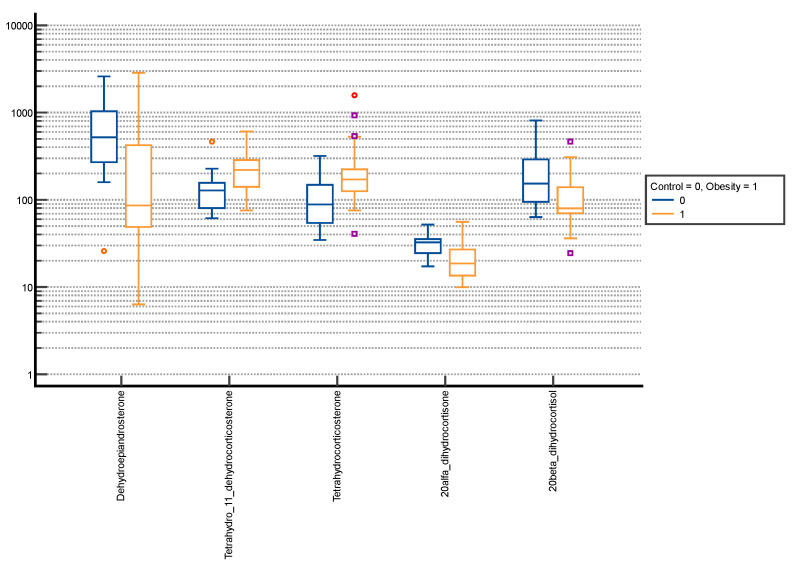
Comparative box plots of steroid metabolites with statistically significant differences (*p*-value < 0.05) between the normal weight and obesity boys in the advanced puberty group. A logarithmic scale was used. Exact results are available in Supplementary Table S2. Purple square—outliers; red circle—extreme values.

**Figure 4:**
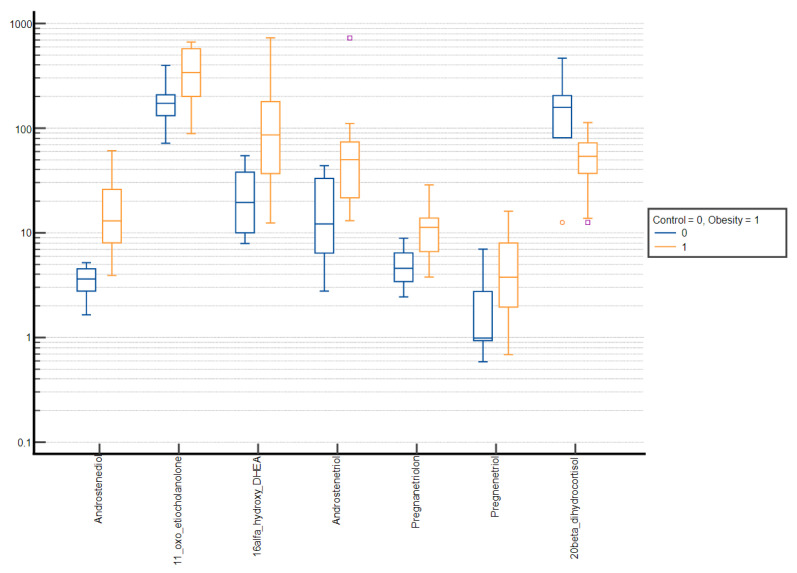
Comparative box plots of steroid metabolites with statistically significant differences (*p*-value < 0.05) between the normal weight and obesity girls in the prepubertal group. A logarithmic scale was used. Exact results are available in Supplementary Table S3. Purple square—outliers, red circle—extreme values.

**Figure 5:**
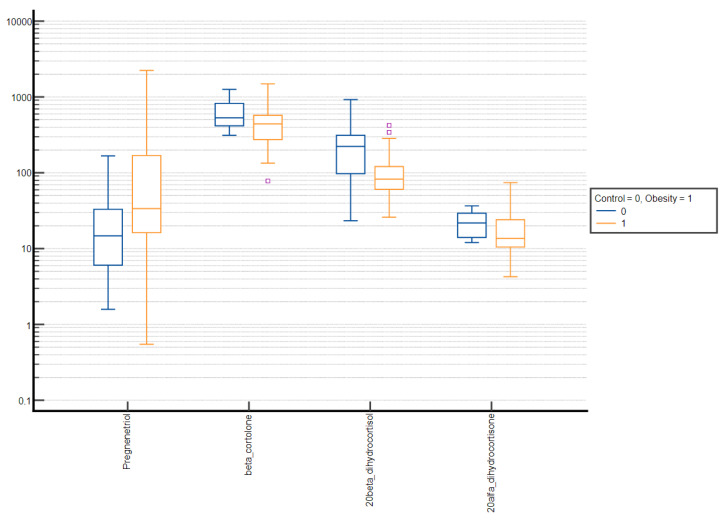
Comparative box plots of steroid metabolites with statistically significant difference (*p*-value < 0.05) between the normal weight and obesity girls in the advanced puberty group. A logarithmic scale was used. Exact results are available in Supplementary Table S4. Purple square – outliers.

Comparative box plots of steroid metabolites displaying statistically significant differences (*p*-value < 0.05) between groups stratified by sex and puberty stage were also performed (Figure 2, Figure 3, Figure 4 and Figure 5).

Finally, to determine the best distinguishing factor to differentiate between obesity and controls, urinary steroid metabolites were assessed using the AUC under the ROC curve for each group (Supplementary Figure S1).

In our study, numerous statistically significant differences were observed between groups. The children with obesity were characterized by increased steroid excretion from all pathways, i.e., mineralo- (tetrahydrodeoxycorticosterone (THDOC), tetrahydro-11-dehydrocorticosterone (THA), tetrahydrocorticosterone (THB), and 5α-tetrahydrocorticosterone (5αTHB)) and glucocorticoids (tetrahydrocortisol (THF), 5α-tetrahydrocortisol (5αTHF), α-cortolone (αCl), α-cortol (αC), β-cortol (βC), and 20β-dihydrocortisone (20βDHE)) as well as adrenal androgens (androsterone (An), etiocholanolone (Et), androstenediol (A5), 5α-dihydrotestosterone (5αDHT), testosterone (T), 11β-hydroxyandrosterone (11β-OH-An), 16α-hydroxy-DHEA, and androstenetriol (AET)) (Table 2). Higher, but not significant, levels of dihydroandrosterone (3αDIOL), dehydroepiandrosterone (DHEA), 11-oxo-etiocholanolone (11-oxo-Et), 11β-hydroxyetiocholanolone (11β-OH-Et), tetrahydro-11-deoxycortisol (THS), tetrahydrocortisone (THE), β-cortolone (βCl), and cortisol (F) were also observed in the study group. Only the excretion of 20α-dihydrocortisone (20αDHE) and 20β-dihydrocortisol (20βDHF) was higher in the control group, but statistical significance was achieved uniquely for 20βDHF.

After stratifying the groups by sex, it turned out that more statistically significant changes were observed when we compared normal weight boys with boys with obesity (Table 3 and Table 4). Similarly to the entire group, changes in the excretion occurred in all three steroid pathways; however, statistical differences for the excretion of An, T, THA, 5αTHB, α-Cl, and 20αDHF in boys were even more pronounced (*p*-values < 0.001). Of note, differences were seen primarily before the onset of puberty (Supplementary Tables S1 and S2). On the contrary, only marginal differences were observed in the group of girls, regardless of their puberty stage. Before puberty, statistically significant differences were observed mainly with regard to DHEA metabolites (A5, 16α-hydroxy-DHEA, and AET), which are excreted in greater amounts by girls with obesity (Supplementary Table S3). In the advanced puberty group, the difference in excretion of cortisol metabolites (βCl, 20αDHE, and 20βDHF) was intensified in favor of the slim female participants (Supplementary Table S4). Therefore, despite the fact that the girls’ cohort in this study was more numerous, it seems that the general results were mainly affected by the changes detectable in male patients. Statistical analysis of the ROC curve indicated 17-hydroxypregnanolone as the best differentiator of alterations in steroid metabolites between normal weight and obesity in the prepubertal boys, while in girls it was androstenediol. In the advanced puberty group, the best indicator to differentiate between normal and excessive body mass was found to be 20α-dihydrocortisone in boys and 20β-dihydrocortisol in girls (Supplementary Figure S1).

## 4. Discussion

### 4.1. General Remarks

In this study, we demonstrated significant differences in the excretion of adrenal steroid metabolites between normal weight children and their peers with obesity. With the steroid profile, it became possible to detect differences between the groups that would not have been noticed if only cortisol, cortisone, or testosterone were measured individually. Additionally, we found significant prepubertal sex differences. Puberty appears to have a pronounced effect on steroid metabolite excretion in boys and limited influence in girls. Body weight increases with age in slim children and even more in children with obesity. The growth process is associated with an increased demand for steroids from the adrenal glands, therefore every older person will typically display enhanced steroid production and subsequent steroid excretion (in absolute terms). However, excessively gained fat tissue may exert some extra influence on this process as well as affect steroid metabolism. Half a century ago, attention was drawn to plausible abnormalities in metabolism of steroids, mainly glucocorticoids, in children with obesity. As noted in the introduction, divergent findings have been reported to date [17,18,19,20,21,22,23,24,29,30,31].

Body mass index is a widely used screening tool in obesity assessment. Using the BMI percentile is recommended in all children once a year. However, BMI cannot differentiate between fat mass (FM) and fat-free mass (FFM). Pruszkowska-Przybylska et al. demonstrated a positive correlation between salivary cortisol and muscle mass and a negative one with body fat mass, although no significant difference of the cortisol concentration was found between children with normal and excessive BMI [38]. The relationship of the steroid profile to body mass composition is a very interesting issue. We plan to evaluate this in future studies to be even more precise than relying on BMI alone, which may result from high muscle mass as well.

Essentially, in our study we found increased excretion of almost all of the investigated metabolites. This can be explained by simple retention of these hormones in the abundant adipose tissue and, consequently, their increased metabolism [39]. Steroids are a heterogeneous group of typically hydrophobic compounds characterized by a tetracyclic fused-ring core; therefore, they are lipophilic and dissolve easily in fats [40]. They probably may “return” from adipose tissue into the circulation when current adrenal steroid secretion decreases in the natural diurnal rhythm. Moreover, features of modern society, including the western diet and the availability of highly processed foods along with increasing environmental stress, may contribute to dysregulation of the HPA axis [13]. Adrenocorticotropic hormone (ACTH) primarily impacts the early, common steps of adrenal steroidogenesis and, subsequently, predominantly the glucocorticoid pathway, to a lesser extent the adrenal androgens, and, negligibly, the mineralocorticoids. Thus, an overactive HPA axis affects all steroid pathways. However, glucocorticoids are the main hormones influencing adipogenesis, adipose metabolism, and promoting fat deposition and central adiposity [9]. It appears that excessive activity of the HPA axis leads to an increase in cortisol concentrations, also within the adipose tissue, followed by an enhanced local lipogenesis and an increase in the amount of fat tissue, which in turn becomes storage for steroid hormones. This is associated with further disturbance in steroid metabolism and closes the vicious circle of the local steroid disorders found in adipose tissue in individuals with obesity. This could potentially explain the difficulties with weight loss in a huge number of people. Moreover, enhanced activity of 11β-hydroxysteroid dehydrogenase type 1 (11βHSD1) has been detected in the fat tissue of obese patients, inducing local cortisol excess (tissue-Cushing) [41,42,43]. Therefore, a selective inhibitor of 11βHSD1 has been suggested as a plausible drug, reducing local cortisol concentrations in various organs, also within the adipose tissue [44,45].At the same time, adipose tissue itself remains an active endocrine organ, and the concentration of its hormones, such as leptin and adiponectin, can directly or indirectly affect steroid metabolism. In mice, leptin modulates the functioning of the HPA axis through its hypothalamic receptor and limits stress-responsive CRH secretion [25,46]. Furthermore, there is some evidence that leptin can regulate human adrenal function directly, through its receptors on adrenocortical cells [25,26,46]. Leptin inhibits ACTH-stimulated steroid release by all three zones within the adrenal glands, with the most severe reduction in cortisol production [26,46]. This seems to be a protective mechanism, as opposed to the local situation in visceral adipose tissue, where glucocorticoid receptors and 11βHSD1 become upregulated in obesity. It has been demonstrated that in vitro adiponectin administration acutely reduces basal corticosterone production and ACTH-induced steroidogenesis in mouse adrenal cortex cells [47]. It can be presumed that a decrease in its concentration in obesity will have the opposite effect. Moreover, epidemiological studies have found that low levels of adiponectin are associated with non-alcoholic steatohepatitis (NASH) [48,49,50]. Liver disease may in turn affect circulating glucocorticoids, although available data are contradictory. It appears that reduced levels of adiponectin in obesity may contribute to liver steatosis and fibrosis, and thus to impaired steroid metabolism [51]. Children in our study had not been diagnosed with alcoholic fatty liver or steatohepatitis, although a lack of clinical manifestation cannot exclude functional changes in the liver, which is the site of intensive steroid metabolism.

The approach to dysregulation of the secretory function and metabolism of steroid hormones should be focused mainly on elimination of excessive body weight, which is the cause and/or effect of steroid disorders. Treatment of childhood obesity should include a combination of lifestyle modifications, as well as nutrition and physical activity behavior changes. Strategies should be implemented to reduce energy intake by changing early infant feeding practices and older children’s eating habits, increasing physical activity, and reducing sedentary lifestyles—including time spent in front of the TV and computer/phone—and improving family involvement.

### 4.2. Prepubertal Results

At the prepubertal stage, numerous statistically significant differences in the excretion of steroid metabolites were detectable in children with obesity compared with their normal weight peers in our study. More alterations were observed in boys than in girls. No significant differences in the amount of excreted free forms of cortisol and cortisone were found, whereas their tetrahydro-derivatives were more abundant in obese children. Tetrahydro-derivatives are irreversible products that may either be eliminated with urine or undergo further unidirectional conversion to cortols and cortolones. In the pre-adolescent children with obesity, they appeared to be excreted more abundantly in the forms of α-cortol and especially α-cortolone than β-cortol or β-cortolone. This may indicate a metabolic preference and skewing towards 20α-hydroxysteroid dehydrogenase (20αHSD). The biological relevance of this enzyme in F and E balance or interconversion remains unknown. The significant increase in multiple cortisol derivatives in the urine suggested the need for more efficient metabolism of larger amounts of F itself, and confirmed previous reports of elevated cortisol production in children with obesity [17,18,19]. Although cortisol levels were not increased compared with healthy controls in our analysis, amplified urinary excretion of its metabolites might reflect enhanced cortisol synthesis and turnover in individuals with obesity. After dividing the prepubertal group by sex, statistical significance in the excretion of F and E derivatives was lost, but, in absolute value, much higher excretion was still maintained in children with obesity, both in girls and boys.

**Figure 6:**
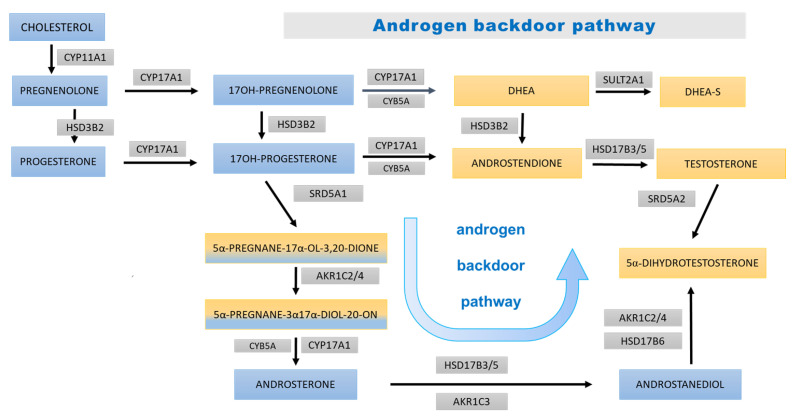
Androgen backdoor steroidogenesis pathway. The blue boxes bear the names of the steroid precursors at early stages of steroidogenesis and for 17OHP and its derivatives; yellow areas (and mixed yellow/blue) are background for androgens and their precursors. The bluish arrows represent steps of the androgen backdoor pathway. The abbreviations of the enzymes involved in each step are displayed in gray boxes: CYP11A1—cytochrome P450 cholesterol monooxygenase (side-chain-cleaving); HSD3B2—3β-hydroxysteroid dehydrogenase type 2; CYP17A1—cytochrome P450 17α-hydroxylase/17,20-lyase; CYB5A—cytochrome b; SULT2A1—sulfotransferase 2A1 (DHEA sulfotransferase); HSD17B3/5—17β-hydroxysteroid dehydrogenase type 3/type 5; SRD5A1 steroid 5α-reductase type 1; AKR1C2/4—aldo-keto reductase 1C2/1C4 (3αHSD); AKR1C3—aldo-keto reductase 1C3 (17β-hydroxysteroid dehydrogenase type 5); HSD17B6—17β-hydroxysteroid dehydrogenase type 6; and SRD5A2 steroid 5α-reductase type 2.

No significant changes were found in the excretion of sex hormones in the girl group, while boys with obesity showed increased excretion of androgens, including testosterone and 5α-dihydrotestosterone. In addition, enhanced insulin resistance secondary to obesity can contribute to the reduction in sex hormone binding globulin (SHBG) concentration in the blood [52]. Considering that SHGB levels in boys and adolescents with excess body mass are lower than in their normal weight peers, and that more than half of total testosterone (TT) binds to this protein, an increase in free testosterone (FT) compared with controls seems to be an expected finding [53]. Moreover, in their study, Cao et al. revealed that changes in FT concentration during growth were higher in the obesity group than in controls at the prepubertal stage, in children aged between 6 and 10 years old, and lower at late puberty. This trend continued at the post-pubertal stage [54]. A considerable difference in 5αDHT levels in pre-adolescent boys with and without excess body mass is noteworthy. The role of 5α-reductase appears to be important, especially in pre-adolescent boys. Enhanced activity of this enzyme may be a starting point for an earlier adrenarche, which is more commonly observed in children with obesity, but can also contribute to earlier puberty due to increased pulsatile GnRH secretion induced by elevated androgens [55,56,57]. Furthermore, we found a significantly elevated excretion of androsterone and etiocholanolone in prepubertal boys with obesity. No differences were found for the studied girls. In contrast to etiocholanolone, which originates almost exclusively from the classic pathway, androsterone additionally may arise from the backdoor androgen pathway, for which it is a characteristic metabolite, and its prevalence in the urine may indicate increased activity of this track (Figure 6) [58]. Recent studies demonstrated a significant association between concurrent obesity indicators, including body fat index with serum dehydroepiandrosterone sulfate (DHEAS), in prepubertal children [59]. Kim et al. showed that body fat mass was positively correlated with serum pregnenolone, dehydroepiandrosterone, androstenedione, testosterone, and androsterone in prepubertal girls [60]. However, it is also true that no significant correlation was reported between DHEAS excretion and concurrent BMI/body fat mass [55,61]. On the other hand, a study from 2009 showed that fat mass was positively associated with adrenal androgen secretion, including DHEA, and explained 5% of its variation. A trend was seen only for the association between FFM and the sum of urinary C19 steroid metabolites (ΣC19), which was much weaker than that between FM and ΣC19 [62]. It seems that different prepubertal impacts of obesity on steroid metabolism in girls and boys may depend on its earlier programming. We may speculate about androgens priming in the fetal life and in the course of mini-puberty experienced only by boys as a plausible pre-activation stage for some enzymes. Girls up to their adrenarche are uniquely exposed to estrogens, which are not converted back to androgens. Therefore, further research should explore these sex differences. Body fat mass has been reported to be closely related to systemic leptin levels in prepubertal children [63]. Kim et al. speculated that leptin produced by adipose tissue might mediate increased steroidogenic enzyme activities in obese children [60].

Regarding the excretion of mineralocorticoid metabolites, there is some former information in the literature about corticosterone (CORT) and its derivatives in obesity, but most reports refer to increased urinary aldosterone (ALD) excretion, mainly in adults [64,65,66]. The relationship between excessive adipose tissue and elevated circulating aldosterone, which leads to the development of hypertension, is well established [67,68,69,70]. Recent data suggest that adipocytes may equally serve as a source of aldosterone, either directly or indirectly, through the release of potential aldosterone-stimulating factors [71,72,73]. Furthermore, Huby et al. hypothesized that leptin is a direct regulator of aldosterone synthase expression and aldosterone release [73]. In our study, we found a significant rise in the excretion of three major corticosterone metabolites: tetrahydro-11-deoxycorticosterone, tetrahydrocorticosterone, and 5α-tetrahydrocorticosterone among prepubertal children with obesity. The effect was maintained in boys, but not in girls. In preclinical models, estrogen has been shown to decrease serum and tissue angiotensin-converting enzyme (ACE) expression, tissue receptor for angiotensin 1 (AT1R), and aldosterone production, while testosterone conversely increases ACE activity and tissue AT1R expression. In contrast to male rodent models of obesity and diabetes, females are protected from metabolic and cardiovascular derangements produced by angiotensinogen, renin, and angiotensin II [74]. This may explain the higher prevalence of hypertension in boys, although no other clinical associations with steroid excretion have been described to date [75,76,77]. Unfortunately, we did not test aldosterone itself. Increased excretion of the above-mentioned corticosterone metabolites indicates a stimulation of the mineralocorticoid pathway. We speculate that obesity-associated hyperleptinemia is a likely responsible factor, as leptin may act directly on adrenal glomerular cells to increase CYP11B2 expression and enhance aldosterone production through calcium-dependent mechanisms [73].

On clinical examination, girls with obesity present more steroid-related abnormalities such as hyperandrogenism and striae, which are not that frequent in boys [78]. Likewise, the manifestation of premature adrenarche is about 10 times more common in girls than in boys [55,79]. Meanwhile, it appears that we found more relevant steroid abnormalities in boys with excessive body mass.

### 4.3. Pubertal Results

In the pubertal cohort, the number of significant differences between children with obesity and their healthy peers was much smaller. Surprisingly, some of glucocorticoid metabolites were even secreted at slightly lower levels in the group of children with obesity compared with normal weight children. In late 1970s, a series of experimental studies in rats revealed significant changes in corticosterone metabolism depending on puberty or gonadectomy and sex steroid replacement. Generally, in rats, puberty and sex steroids decrease the activity of 5α-reductase, which may be then restored after gonadectomy in both sexes [80,81]. These observations seem to be in line with the changes in excreted metabolites demonstrated in our study, along with the progress of puberty. This phenomenon may be additionally modified by coexisting obesity. According to Wudy et al., cortisol secretion in normal weight individuals shows a dynamic pattern throughout childhood and adolescence, with periods of more rapid growth and decline, while urinary cortisol remains relatively constant [82]. We assume that children with obesity may also exhibit dynamic changes in cortisol excretion and its derivatives, but their pattern across puberty may be altered if compared with subjects without obesity.

Androgen assessment in the group with advanced puberty is difficult, because adrenal androgenic activity becomes obscured by increasing sex steroid synthesis in gonads. Furthermore, it is well known that sex steroid hormones alter the body composition during pubertal development. Estrogen, for example, is known to play a crucial role in body fat distribution [83]. This difference in hormone actions leads to more increased lean mass in boys, and comparatively high fat mass in girls [84]. Ridder et al. demonstrated that body fat distribution, rather than body fat mass, is different in relation to the total concentrations of estrone, estradiol, and testosterone in pubertal girls and young females [85,86].

A decrease in the excretion of androgen metabolites (An, Et, DHEA, T, and 5αDHT) was observed in the group of boys with advanced puberty, which remains in accordance with earlier reports of a drop in T level in boys with obesity during puberty [53,54,87,88,89]. Elevated serum leptin levels that persist for several years of excessive fat content may also be responsible for this effect [90,91,92]. In girls, we observed significantly higher excretion of pregnenetriol among adolescents with obesity, which, for instance, might explain the common presence of idiopathic hirsutism in this group [93,94,95,96].

In the group with advanced puberty, the pattern of increased urinary mineralocorticoid secretion, observed from the prepubertal period, still persisted. This was especially noticeable in boys, in whom statistically significant differences in THA and THB excretion between adolescents with and without obesity were found.

Our current study displays several limitations that need to be addressed. The COVID-19 pandemic limited our ability to collect more participants in the study group and in the control cohort. Moreover, further stratification of the studied cohorts by sex and puberty stage resulted in even smaller subgroups, which definitely limited the power of the study to detect more subtle differences in steroid excretion. We selected children before puberty, but puberty itself can also proceed differently in particular individuals and hence could have affected outcomes across the whole group. Our conclusions may not be complete because we evaluated the metabolites in urine, but not at the point of their formation, so we did not have a comprehensive insight into what happened “along the way”. On the other hand, the number of metabolites determined in urine exceeded that in blood and provided us with more extensive information. Finally, our observations and their interpretation must take into account that the metabolic status in children and adolescents is not as stable and homogeneous as in adults with obesity. However, early-onset obesity is of particular research interest, as it may be associated with some inherent metabolic features, which promote its early development, and potentially increase susceptibility for future complications. Further research and evaluation of metabolites, as well as assessment of the activity of specific enzymes, should improve the understanding of steroid disorders in individuals with obesity.

## 5. Concluding Remarks

Children with excessive body mass seem to display multiple subtle dysregulations in steroid synthesis, metabolism, and excretion. They present significantly higher urinary elimination of several steroid metabolites compared with their normal weight peers. Obesity in youth is associated with enhanced activities of all the adrenocortical steroidogenic pathways, as reflected by increased excretion of the mineralocorticoid derivatives (tetrahydro-11-deoxycorticosterone, tetrahydrocorticosterone, and 5α-tetrahydrocorticosterone), tetrahydro-derivatives of cortisol/cortisone (α-cortol and especially α-cortolone), and elevated androgen excretion with an apparently enhanced backdoor pathway compared with slim coevals. Urine excretion of estrogens is independent of body mass and correlates with age, sex, and puberty stage. Furthermore, activation of the adrenal cortex, together with concomitant increase in steroid excretion, is more prominent in boys with obesity than in girls. Most of the observed differences between excess and normal weight children are particularly pronounced in the prepubertal period, and then decline as puberty progresses. Further studies are warranted to elucidate the meaning of changes in the steroid metabolism in obesity. The major challenge remains to distinguish the effects of increased adrenocortical secretion from the results of steroid sequestration in adipose tissue or its metabolism in the liver, as well as in other body organs. It also seems promising to look at glucocorticosteroids, as hormonal signals of appetite and disturbances in their concentration may be future biomarkers and treatment targets for obesity, regardless of age.

## Figures and Tables

**Table 1:** Clinical phenotype of normal weight and obesity groups.

	Normal Weight (n = 76)	Obesity (n = 115)	*p* Value
Sex (male/female)	41/35	51/64	0.195
	Median (IQR)	Median (IQR)	
Age (years)	11.5 (8–13)	12 (9–14)	0.051
BMI (kg/m^2^)	17.10 (15.55–18.86)	28.93 (26.02–32.78)	<0.001
z score BMI/SDS BMI	0.02 (−0.72–0.43)	2.20 (1.90–2.50)	<0.001
	Mean ± SD	Mean ± SD	
Body surface area (m^2^)	1.27 ± 0.32	1.83 ± 0.41	<0.001

Statistically significant differences are marked in red.

**Table 2:** List of steroid metabolites (μg/24 h): differences between the normal weight and obesity groups.

	Normal Weight (n = 76)		Obesity (n = 115)		*p* Value
	Mean ± SD	Median (IQR)	Mean ± SD	Median (IQR)	
Androsterone	845.80 ± 890.07	450.63 (141.95–1386.35)	1279.66 ± 1171.43	996.49 (429.78–1792.42)	0.001
Etiocholanolone	591.47 ± 738.17	343.10 (107.88–660.85)	693.45 ± 647.57	506.89 (241.14–955.38)	0.015
Dihydroandrosterone	28.49 ± 23.68	21.88 (13.22–33.34)	41.34 ± 38.52	28.34 (13.03–55.14)	0.056
Dehydroepiandrosterone	176.47 ± 253.68	51.41 (18.21–235.71)	188.49 ± 344.23	58.80 (22.97–156.85)	0.895
Androstenediol	18.55 ± 18.92	11.40 (4.63–25.72)	49.00 ± 67.49	30.37 (14.92–59.42)	<0.001
11-oxo-etiocholanolone	392.08 ± 279.23	311.45 (213.12–490.92)	443.61 ± 316.10	354.42 (209.57–629.06)	0.341
5α-dihydrotestosterone	11.16 ± 9.37	8.33 (4.74–14.27)	14.91 ± 12.69	11.74 (5.39–18.62)	0.047
17β-estradiol	0.86 ± 0.93	0.56 (0.26–1.08)	1.40 ± 1.32	1.01 (0.40–1.99)	0.006
Testosterone	16.66 ± 17.28	10.89 (6.55–18.07)	21.85 ± 18.28	16.14 (9.05–31.01)	0.007
11β-hydroxyandrosterone	656.84 ± 451.76	521.4 (311.87–888.41)	889.12 ± 642.59	755.95 (415.01–1243.58)	0.012
11β-hydroxyetiocholanolone	188.11 ± 195.92	124.13 (46.39–239.81)	240.42 ± 213.05	184.57 (75.47–333.51)	0.056
17-hydroxypregnanolone	13.17 ± 14.57	7.73 (3.76–14.92)	18.96 ± 16.42	14.44 (7.61–24.09)	<0.001
16α-hydroxy-DHEA	167.09 ± 188.99	74.55 (38.71–209.44)	325.20 ± 340.57	211.16 (75.37–482.19)	<0.001
Pregnanediol	110.41 ± 126.58	62.30 (38.99–122.43)	239.98 ± 251.82	155.29 (65.84–318.51)	<0.001
Pregnanetriol	310.12 ± 293.26	196.82 (109.49–359.81)	401.44 ± 262.69	329.16 (192.70–546.69)	<0.001
Androstenetriol	135.31 ± 148.68	72.61 (34.14–185.76)	231.29 ± 256.07	134.13 (39.82–359.94)	0.016
Tetrahydro-11-deoxycortisol	58.73 ± 35.64	46.55 (33.14–75.32)	57.63 ± 39.59	50.06 (28.13–81.68)	0.695
Tetrahydrodeoxycorticosterone	4.79 ± 3.60	3.46 (2.50–5.91)	6.66 ± 5.69	4.51 (3.02–8.63)	0.017
Estriol	1.67 ± 2.00	1.14 (0.34–2.22)	3.22 ± 4.43	1.45 (0.52–3.91)	0.035
Pregnanetriolone	11.12 ± 8.24	8.63 (5.56–14.63)	13.78 ± 10.56	10.51 (6.81–16.37)	0.021
Pregnenetriol	18.10 ± 31.88	5.85 (2.85–20.68)	59.16 ± 121.42	15.79 (5.42–38.89)	<0.001
Tetrahydrocortisone	2371.15 ± 1146.68	2161.63 (1558.55–2992.66)	2720.05 ± 1667.70	2372.18 (1541.86–3648.83)	0.303
Tetrahydro-11-dehydrocorticosterone	122.62 ± 75.87	102.48 (64.13–148.94)	163.75 ± 106.48	136.53 (81.84–226.31)	0.008
Tetrahydrocorticosterone	72.84 ± 42.60	59.68 (41.91–93.35)	144.18 ± 113.52	113.08 (68.76–178.99)	<0.001
5α-tetrahydrocorticosterone	289.81 ± 169.52	252.70 (161.16–385.92)	389.78 ± 266.51	336.39 (183.71–526.18)	0.022
Tetrahydrocortisol	721.02 ± 420.19	592.07 (443.3–911.84)	1021.99 ± 706.78	850.32 (494.23–1484.35)	0.006
5α-tetrahydrocortisol	1011.64 ± 590.83	833.27 (549.55–1299.59)	1332.93 ± 864.83	1099.11 (689.64–1807.75)	0.015
α-cortolone	1004.45 ± 604.63	913.48 (513.79–1418.05)	1361.10 ± 778.73	1186.50 (776.58–1886.82)	0.001
β-cortolone	382.01 ± 176.54	349.27 (271.85–452.78)	430.64 ± 246.56	377.28 (251.96–563.77)	0.384
α-cortol	184.50 ± 107.48	163.35 (98.24–236.65)	272.91 ± 194.44	235.22 (123.49–382.65)	0.002
β-cortol	292.23 ± 151.55	267.11 (186.21–353.62)	396.11 ± 251.42	314.35 (198.22–512.97)	0.010
Cortisone	105.78 ± 44.98	95.19 (74.54–129.84)	107.96 ± 59.26	93.87 (63.20–146.34)	0.724
Cortisol	111.13 ± 51.77	95.76 (75.73–139.24)	117.29 ± 70.74	108.28 (66.51–154.64)	0.835
20α-dihydrocortisone	17.84 ± 8.76	14.69 (11.05–23.04)	15.61 ± 8.37	13.45 (9.91–20.08)	0.085
20β-dihydrocortisone	48.01 ± 27.76	37.19 (26.62–65.76)	70.63 ± 43.62	60.75 (39.80–96.14)	<0.001
20α-dihydrocortisol	31.40 ± 21.39	23.85 (15.21–44.24)	60.73 ± 63.58	37.0 (22.71–72.40)	0.001
20β-dihydrocortisol	162.61 ± 113.96	136.18 (74.06–242.47)	90.75 ± 60.33	75.17 (55.01–105.95)	<0.001
6β-hydroxycortisol	40.12 ± 20.41	35.65 (25.25–47.90)	37.96 ± 27.82	33.12 (16.66–47.63)	0.098

Statistically significant differences are marked in red.

**Table 3:** List of steroid metabolites (μg/24 h): differences between the normal weight and obesity in boys.

	Normal Weight (n = 41)		Obesity (n = 51)		*p* Value
	Mean ± SD	Median (IQR)	Mean ± SD	Median (IQR)	
Androsterone	653.13 ± 803.43	282.87 (87.38–866.49)	1356.86 ± 1307.30	1028.14 (457.14–1575.73)	<0.001
Etiocholanolone	485.24 ± 693.07	152.90 (54.78–566.72)	650.52 ± 640.08	429.14 (270.90–696.39)	0.008
Dihydroandrosterone	30.99 ± 24.02	24.58 (15.51–36.72)	44.07 ± 37.02	29.87 (11.89–60.15)	0.154
Dehydroepiandrosterone	230.36 ± 280.89	122.47 (40.09–269.88)	167.99 ± 236.94	64.00 (25.58–189.07)	0.134
Androstenediol	13.94 ± 15.42	7.52 (3.94–17.11)	45.03 ± 59.82	31.39 (14.71–58.17)	<0.001
11-oxo-etiocholanolone	409.79 ± 271.85	323.25 (262.54–493.25)	497.65 ± 368.65	462.34 (179.27–666.90)	0.369
5α-dihydrotestosterone	11.87 ± 11.07	7.78 (4.47–13.40)	17.78 ± 13.66	14.71 (8.73–18.62)	0.002
17β-estradiol	0.66 ± 0.79	0.33 (0.19–0,83)	1.15 ± 1.01	0.93 (0.45–1.62)	0.006
Testosterone	17.52 ± 19.69	8.20 (6.23–17.56)	28.76 ± 21.21	22.67 (11.99–39.29)	<0.001
11β-hydroxyandrosterone	570.48 ± 419.82	430.25 (255.67–710.36)	951.00 ± 640.34	794.23 (457.24–1374.93)	0.001
11β-hydroxyetiocholanolone	181.18 ± 165.77	116.51 (68.01–264.51)	258.74 ± 217.19	209.98 (93.13–347.41)	0.846
17-hydroxypregnanolone	11.83 ± 13.99	6.01 (3.17–14.07)	19.66 ± 15.51	16.20 (8.00–25.21)	<0.001
16α-hydroxy-DHEA	184.53 ± 206.65	75.50 (50.25–219.01)	332.24 ± 309.51	216.51 (85.78–534.38)	0.009
Pregnanediol	75.76 ± 97.04	50.42 (22.67–93.72)	220.95 ± 222.41	160.36 (68.86–281.56)	<0.001
Pregnanetriol	264.78 ± 290.62	161.12 (88.11–291.54)	385.38 ± 271.80	306.26 (204.64–498.22)	<0.001
Androstenetriol	109.19 ± 129.82	61.74 (26.42–149.43)	215.18 ± 239.55	135.05 (36.18–269.45)	0.029
Tetrahydro-11-deoxycortisol	55.54 ± 39.58	42.15 (24.29–72.07)	56.63 ± 43.65	49.59 (20.46–91.03)	0.859
Tetrahydrodeoxycorticosterone	3.96 ± 2.97	3.40 (1.93–4.63)	6.75 ± 4.93	5.25 (3.27–8.74)	0.001
Estriol	1.01 ± 1.23	0.54 (0.25–1.33)	1.87 ± 2.06	1.29 (0.52–2.03)	0.013
Pregnanetriolone	11.59 ± 9.19	8.60 (5.27–15.09)	15.08 ± 8.57	12.11 (7.95–21.86)	0.007
Pregnenetriol	16.93 ± 28.70	5.85 (3.08–21.58)	51.89 ± 114.63	16.26 (6.23–38.34)	<0.001
Tetrahydrocortisone	2216.66 ± 1140.52	1893.05 (1342.96–2665.82)	2892.98 ± 1848.39	2485.20 (1539.14–4126.22)	0.162
Tetrahydro-11-dehydrocorticosterone	110.72 ± 68.27	92.03 (59.35–140.44)	198.45 ± 119.34	165.07 (85.75–269.29)	<0.001
Tetrahydrocorticosterone	65.96 ± 40.18	50.58 (38.73–84.21)	170.96 ± 102.09	166.37 (105.61–213.84)	<0.001
5α-tetrahydrocorticosterone	269.23 ± 187.98	217.74 (150.55–319.23)	461.15 ± 297.05	386.10 (227.10–692.72)	<0.001
Tetrahydrocortisol	686.75 ± 410.34	588.34 (408.85–876,87)	1023.41 ± 697.91	877.25 (495.11–1493.72)	0.021
5α-tetrahydrocortisol	1013.30 ± 679.39	727.36 (525.08–1400.68)	1369.13 ± 899.68	1082.87 (810.26–1687.00)	0.028
α-cortolone	828.45 ± 538.89	680.93 (455.89–1069.10)	1529.24 ± 899.47	1315.29 (782.57–2211.02)	<0.001
β-cortolone	352.96 ± 144.16	337.57 (267.95–421.68)	469.75 ± 265.91	435.00 (254.44–646.26)	0.067
α-cortol	171.85 ± 108.28	154.34 (85.04–202.52)	296.45 ± 216.01	231.41 (127.20–437.34)	0.004
β-cortol	285.70 ± 142.82	262.60 (185.45–335.85)	411.79 ± 245.49	344.56 (208.11–595.33)	0.022
Cortisone	102.93 ± 48.73	87.54 (66.59–130.07)	114.64 ± 59.41	101.73 (69.27–157.83)	0.376
Cortisol	105.14 ± 51.72	85.89 (69.19–124.61)	121.35 ± 62.58	115.50 (71.60–161.68)	0.160
20α-dihydrocortisone	17.84 ± 9.45	15.22 (10.37–23.78)	17.80 ± 8.54	15.96 (11.56–24.14)	0.830
20β-dihydrocortisone	42.89 ± 26.29	32.65 (23.10–58.10)	79.51 ± 45.29	63.84 (52.71–115.57)	<0.001
20α-dihydrocortisol	30.27 ± 21.73	21.04 (14.93–43.17)	66.84 ± 60.47	48.67 (27.02–82.09)	<0.001
20β-dihydrocortisol	141.13 ± 109.10	108.09 (61.63–175.12)	94.64 ± 54.98	77.27 (61.33–116.12)	0.101
6β-hydroxycortisol	39.18 ± 18.70	35.63 (24.95–48.02)	40.41 ± 25.64	34.23 (23.46–51.37)	0.846

Statistically significant differences are marked in red.

**Table 4:** List of steroid metabolites (μg/24 h): differences between the normal weight and obesity in girls.

	Normal Weight (n = 35)		Obesity (n = 64)		*p* Value
	Mean ± SD	Median (IQR)	Mean ± SD	Median (IQR)	
Androsterone	1066.79 ± 932.27	920.82 (330.86–1631.84)	1216.16 ± 1042.25	990.39 (402.24–1873.63)	0.452
Etiocholanolone	719.57 ± 769.87	447.42 (166.18–713.82)	728.76 ± 651.54	559.42 (192.67–1070.65)	0.534
Dihydroandrosterone	25.70 ± 22.98	20.81 (10.97–30.14)	39.21 ± 39.52	25.47 (13.35–47.04)	0.123
Dehydroepiandrosterone	116.42 ± 203.24	26.09 (13.50–73.53)	204.76 ± 409.12	57.87 (22.64–141.59)	0.051
Androstenediol	23.68 ± 21.02	15.25 (6.25–32.84)	52.22 ± 72.95	26.31 (15.04–59.76)	0.007
11-oxo-etiocholanolone	370.73 ± 286.42	284.79 (182.12–457.45)	403.08 ± 262.87	315.47 (210.59–592.21)	0.382
5α-dihydrotestosterone	10.31 ± 6.67	8.58 (5.52–14.62)	12.67 ± 11.38	7.69 (3.41–18.41)	0.967
17β-estradiol	1.10 ± 1.02	0.93 (0.34–1.27)	1.59 ± 1.49	1.13 (0.38–2.29)	0.296
Testosterone	15.69 ± 14.05	11.46 (7.73–17.98)	16.27 ± 13.09	13.09 (6.77–20.59)	0.802
11β-hydroxyandrosterone	758.44 ± 466.62	717.92 (393.80–947.83)	839.02 ± 640.04	720.87 (383.20–1048.98)	0.800
11β-hydroxyetiocholanolone	196.46 ± 226.73	134.04 (45.14–205.93)	226.46 ± 208.77	175.76 (69.88–274.73)	0.227
17-hydroxypregnanolone	14.69 ± 15.07	10.34 (4.55–17.24)	18.39 ± 17.11	12.27 (7.08–22.78)	0.147
16α-hydroxy-DHEA	145.42 ± 161.83	73.59 (34.59–192.17)	319.51 ± 363.68	203.60 (71.15–344.09)	0.010
Pregnanediol	153.46 ± 144.51	84.99 (56.94–194.70)	255.89 ± 272.98	121.03 (60.05–341.56)	0.118
Pregnanetriol	364.78 ± 287.06	275.16 (147.60–449.71)	414.87 ± 254.04	403.79 (191.49–548.23)	0.174
Androstenetriol	164.41 ± 162.32	117.22 (44.81–234.20)	244.33 ± 268.00	120.99 (55.45–390.61)	0.271
Tetrahydro-11-deoxycortisol	62.47 ± 29.97	54.64 (41.68–82.55)	58.40 ± 36.10	51.19 (35.09–72.99)	0.380
Tetrahydrodeoxycorticosterone	5.70 ± 4.01	3.60 (2.98–7.44)	6.60 ± 6.20	4.36 (2.80–8.32)	0.887
Estriol	2.48 ± 2.42	1.52 (1.00–3.03)	4.39 ± 5.48	2.01 (0.56–6.23)	0.496
Pregnanetriolone	10.53 ± 6.85	8.88 (5.99–12.74)	12.76 ± 11.78	9.59 (6.43–13.53)	0.447
Pregnenetriol	19.43 ± 35.11	5.85 (2.85–14.48)	65.13 ± 126.43	15.27 (4.00–44.33)	0.025
Tetrahydrocortisone	2552.12 ± 1127.24	2246.55 (1762.23–3400.38)	2582.81 ± 1494.81	2371.05 (1651.51–3371.48)	0.819
Tetrahydro-11-dehydrocorticosterone	136.22 ± 81.62	106.18 (71.02–160.85)	136.76 ± 86.11	117.83 (67.93–181.15)	0.994
Tetrahydrocorticosterone	80.94 ± 43.92	67.00 (49.09–99.84)	123.67 ± 117.49	92.40 (51.96–147.67)	0.125
5α-tetrahydrocorticosterone	312.74 ± 142.79	319.87 (187.29–409.20)	332.23 ± 223.00	293.21 (167.32–426.06)	0.813
Tetrahydrocortisol	762.34 ± 428.13	607.95 (452.74–942.51)	1020.84 ± 713.88	793.66 (495.93–1438.85)	0.134
5α-tetrahydrocortisol	1009.79 ± 473.00	916.86 (728.80–1228.78)	1304.32 ± 835.15	1158.05 (573.10–1846.48)	0.142
α-cortolone	1216.69 ± 611.42	1093.47 (825.42–1541.25)	1227.66 ± 636.68	1165.45 (777.78–1598.56)	0.973
β-cortolone	417.04 ± 203.57	356.66 (287.58–515.08)	399.60 ± 225.25	341.12 (237.95–536.46)	0.580
α-cortol	199.75 ± 104.49	181.04 (106.95–246.01)	253.55 ± 172.31	236.48 (119.12–337.55)	0.223
β-cortol	300.35 ± 161.37	277.50 (190.24–359.54)	383.20 ± 255.48	308.58 (183.24–498.16)	0.166
Cortisone	109.04 ± 40.03	108.61 (78.08–126.55)	102.66 ± 58.61	88.70 (62.27–136.47)	0.164
Cortisol	117.98 ± 50.97	104.99 (81.90–148.10)	114.14 ± 76.35	99.96 (60.83–141.61)	0.304
20α-dihydrocortisone	17.84 ± 7.89	14.07 (12.53–22.13)	13.87 ± 7.80	12.15 (9.01–16.27)	0.009
20β-dihydrocortisone	53.85 ± 28.24	45.49 (33.63–69.77)	63.73 ± 40.96	58.96 (37.33–75.50)	0.311
20α-dihydrocortisol	32.80 ± 20.87	24.64 (18.27–44.90)	55.79 ± 65.57	31.89 (20.03–56.11)	0.218
20β-dihydrocortisol	187.87 ± 114.37	174.49 (107.56–262.23)	87.79 ± 63.96	66.59 (51.02–103.82)	<0.001
6β-hydroxycortisol	41.23 ± 22.19	35.68 (27.85–45.96)	36.02 ± 29.29	26.21 (15.79–46.69)	0.061

Statistically significant differences are marked in red.

## Data Availability

The datasets used and/or analyzed during the current study are available from the corresponding author upon request.

## Supplementary Materials

The following supporting information can be downloaded at: https://www.mdpi.com/article/10.3390/nu15071734/s1, Table S1: List of steroid metabolites (μg/24 h): differences between the normal weight and obesity in prepubertal boys; Table S2: List of steroid metabolites (μg/24 h): differences between the normal weight and obesity in boys with advanced puberty; Table S3: List of steroid metabolites (μg/24 h): differences between the normal weight and obesity in prepubertal girls; Table S4: List of steroid metabolites (μg/24 h): differences between the normal weight and obesity in girls with advanced puberty; Figure S1: Results of the analysis of the ROC curve for most appropriate classifier to distinguish alterations in steroid metabolites between normal weight and obesity. The blue shaped area is the 95% confidence interval of the sensitivity at the given specificity. (A) whole group divided by sex, (B) prepubertal group divided by sex, (C) group with advanced puberty divided by sex.
