# Supplementary material for: The Impact of Obesity on the Excretion of Steroid Metabolites in Boys and Girls: A Comparison with Normal-Weight Children
Source: Nutrients. 2023 Apr 1;15(7):1734. doi: 10.3390/nu15071734 (PMC10097123; doi:10.3390/nu15071734)
Supplement: Supplementary file 1 [file nutrients-15-01734-s001.zip › nutrients-2272314-supplementary.pdf]

Supplementary Materials

Supplementary Table S1. List of steroid metabolites (μg/24h): differences between the normal weight and obesity in prepubertal boys

	Normal weight (n=18)		Obesity (n=16)		*p* value
	Mean+SD	Median (IQR)	Mean+SD	Median (IQR)	
Androsterone	98.43 ± 77.31	80.35 (38.08-125.36)	467.63 ± 417.04	265.81 (172.85-631.27)	<0.001
Etiocholanolone	69.51 ± 51.76	54.62 (27.48-98.83)	233.07 ± 175.46	141.01 (104.03-342.32)	<0.001
Dihydroandrosterone	19.20 ± 15.61	15.39 (6.89-25.29)	23.77 ± 35.73	10.34 (7.20-25.42)	0.798
Dehydroepiandrosterone	105.39 ± 74.41	108.30 (50.62-141.13)	113.24 ± 168.37	42.93 (23.04-107.71)	0.330
Androstenediol	5.38 ± 3.91	5.18 (2.27-7.60)	23.38 ± 24.53	12.96 (5.27-35.04)	0.005
11-oxo-etiocholanolone	283.59 ± 121.12	301.12 (196.48-348.76)	421.38 ± 371.97	334.77 (105.59-618.21)	0.605
5α-dihydrotestosterone	5.41 ± 3.30	4.23 (2.97-7.57)	20.16 ± 27.50	14.36 (7.38-16.82)	0.005
17ß-estradiol	0.72 ± 0.75	0.40 (0.10-1.08)	1.16 ± 0.95	0.99 (0.25-1.85)	0.198
Testosterone	6.78 ± 2.25	6.24 (5.96-7.70)	13.06 ± 11.14	9.37 (6.53-16.08)	0.036
11ß-hydroxyandrosterone	259.65 ± 112.38	237.94 (214.01-362.14)	540.32 ± 338.59	426.35 (293.36-785.37)	0.005
11ß-hydroxyetiocholanolone	125.69 ± 89.06	113.73 (55.12-189.91)	257.54 ± 289.73	128.04 (40.18-298.50)	0.486
17-hydroxypregnanolone	3.97 ± 2.73	3.27 (2.40-4.55)	12.88 ± 10.15	8.02 (6.59-17.01)	<0.001
16α-hydroxy-DHEA	67.63 ± 58.63	55.27 (28.85-66.79)	300.24 ± 385.15	99.49 (38.70-394.22)	0.081
Pregnanediol	32.45 ± 29.16	21.77 (13.48-44.23)	191.79 ± 279.53	128.60 (44.97-184.13)	0.001
Pregnanetriol	87.28 ± 41.66	85.71 (50.65-120.93)	217.07 ± 133.06	170.91 (122.03-310.90)	<0.001
Androstenetriol	31.30 ± 27.14	23.46 (6.62-50.60)	56.43 ± 58.95	29.93 (11.34-95.86)	0.330
Tetrahydro-11-deoxycortisol	49.47 ± 33.96	41.48 (23.95-63.78)	65.96 ± 52.51	58.03 (25.88-80.33)	0.486
Tetrahydrodeoxycorticosterone	3.44 ± 2.26	3.20 (2.35-3.72)	6.93 ± 3.90	6.30 (3.37-9.30)	0.003
Estriol	0.48 ± 0.36	0.35 (0.25-0.58)	0.69 ± 0.51	0.73 (0.29-0.99)	0.313
Pregnanetriolone	6.59 ± 3.40	5.69 (4.55-7.77)	16.48 ± 9.57	12.98 (9.90-21.67)	<0.001
Pregnenetriol	4.24 ± 5.99	2.39 (1.07-4.20)	11.65 ± 8.90	7.86 (5.38-15.51)	<0.001
Tetrahydrocortisone	1675.81 ± 877.13	1375.59 (1193.58-1812.31)	2722.70 ± 2076.95	2253.85 (1152.19-3246.49)	0.313
Tetrahydro-11-dehydrocorticosterone	95.62 ± 68.15	65.80 (53.16-112.66)	199.99 ± 150.59	117.24 (81.34-310.30)	0.007
Tetrahydrocorticosterone	51.04 ± 29.68	40.93 (36.46-47.86)	140.22 ± 78.21	132.25 (76.70-216.79)	<0.001
5α-tetrahydrocorticosterone	210.36 ± 186.16	150.55 (128.22-209.06)	422.76 ± 285.85	334.91 (165.55-709.64)	0.033
Tetrahydrocortisol	498.63 ± 244.28	429.82 (345.60-532.27)	730.60 ± 509.15	568.47 (303.59-858.47)	0.251
5α-tetrahydrocortisol	700.12 ± 541.02	531.19 (454.98-691.01)	1152.20 ± 837.95	1049.48 (576.78-1450.30)	0.022
α-cortolone	499.77 ± 286.43	444.14 (337.37-544.27)	1195.25 ± 906.94	912.12 (447.11-1538.73)	0.009
ß-cortolone	283.01 ± 103.51	267.97 (226.85-349.42)	439.39 ± 287.90	377.95 (188.81-598.75)	0.251
α-cortol	105.68 ± 48.13	85.76 (79.88-121.62)	188.82 ± 131.35	148.37 (97.80-261.41)	0.075
ß-cortol	222.29 ± 108.93	220.39 (164.25-260.35)	301.14 ± 207.40	232.52 (154.51-400.88)	0.463
Cortisone	81.61 ± 32.05	77.23 (61.66-90.59)	104.85 ± 77.37	69.76 (46.65-163.92)	1.000
Cortisol	83.36 ± 32.05	75.73 (62.52-87.88)	96.10 ± 56.99	74.79 (55.95-123.86)	0.878
20α-dihydrocortisone	13.42 ± 6.25	11.29 (9.92-17.13)	15.03 ± 9.85	11.54 (7.97-17.82)	0.905
20ß-dihydrocortisone	28.22 ± 14.01	23.50 (19.83-32.59)	68.37 ± 54.91	47.37 (30.17-88.74)	0.010
20α-dihydrocortisol	23.23 ± 17.85	16.10 (12.20-28.03)	41.85 ± 37.37	31.00 (10.97-65.83)	0.422
20ß-dihydrocortisol	126.52 ± 96.80	96.26 (43.75-165.23)	76.46 ± 34.90	62.52 (58.42-88.80)	0.442
6ß-hydroxycortisol	33.41 ± 13.43	31.54 (22.33-43.80)	38.18 ± 27.33	33.48 (15.52-48.13)	0.986

Supplementary Table S2. List of steroid metabolites (µg/24h): differences between the normal weight and obesity in boys with advanced puberty

	Normal weight (n = 12)		Obesity (n = 28)		*p* value
	Mean+SD	Median (IQR)	Mean+SD	Median (IQR)	
Androsterone	2385.34 ± 1602.63	1953.79 (1531.54-2593.81)	1968.90 ± 1426.66	1477.15 (986.05-2717.29)	0.328
Etiocholanolone	1245.88 ± 858.90	1011.44 (626.36-1740.87)	955.41 ± 715.79	587.26 (424.83-1184.96)	0.202
Dihydroandrosterone	78.34 ± 46.93	67.53 (45.68-91.10)	67.20 ± 50.71	56.60 (34.74-80.52)	0.358
Dehydroepiandrosterone	786.77 ± 736.39	521.20 (273.67-971.03)	324.77 ± 558.52	86.32 (49.71-421.15)	0.010
Androstenediol	55.23 ± 47.61	41.53 (22.51-54.05)	58.71 ± 73.89	43.05 (20.82-63.30)	0.919
11-oxo-etiocholanolone	697.89 ± 303.35	712.95 (481.06-844.82)	590.36 ± 468.85	565.74 (253.11-759.14)	0.202
5α-dihydrotestosterone	24.61 ± 11.95	26.44 (16.07-32.87)	20.83 ± 14.91	15.31 (8.92-35.70)	0.389
17ß-estradiol	1.33 ± 1.55	0.75 (0.43-1.08)	1.40 ± 1.44	0.94 (0.48-1.63)	0.610
Testosterone	57.35 ± 36.44	52.41 (23.70-71.40)	48.55 ± 40.84	38.31 (27.40-58.12)	0.475
11ß-hydroxyandrosterone	1232.31 ± 646.76	1171.65 (723.71-1529.15)	1217.40 ± 681.94	1139.65 (640.52-1594.88)	0.942
11ß-hydroxyetiocholanolone	315.69 ± 211.25	266.88 (160.04-444.04)	332.76 ± 300.69	256.86 (115.30-459.12)	0.850
17-hydroxypregnanolone	33.81 ± 30.38	18.65 (13.97-47.37)	24.40 ± 17.36	21.93 (11.43-29.79)	0.610
16α-hydroxy-DHEA	441.16 ± 210.11	381.91 (293.39-561.04)	370.53 ± 275.32	271.84 (150.79-641.50)	0.224
Pregnanediol	155.30 ± 143.97	114.80 (55.22-165.41)	232.12 ± 194.19	189.44 (82.23-276.15)	0.122
Pregnanetriol	589.28 ± 352.02	559.52 (281.61-919.63)	507.04 ± 295.34	393.98 (274.58-696.93)	0.760
Androstenetriol	361.75 ± 236.84	267.99 (207.19-461.82)	312.57 ± 243.53	239.67 (140.75-395.14)	0.512
Tetrahydro-11-deoxycortisol	63.82 ± 44.94	42.92 (39.95-77.30)	62.68 ± 49.50	55.21 (21.93-94.17)	0.850
Tetrahydrodeoxycorticosterone	7.29 ± 6.37	4.98 (3.07-8.40)	7.81 ± 8.40	5.25 (3.25-8.67)	0.805
Estriol	3.12 ± 3.82	1.89 (0.76-3.05)	2.69 ± 2.24	1.72 (1.28-3.38)	0.782
Pregnanetriolone	17.58 ± 10.82	15.91 (9.43-23.30)	14.05 ± 7.58	11.06 (7.80-21.77)	0.493
Pregnenetriol	97.81 ± 178.07	28.84 (21.80-68.30)	74.89 ± 143.71	33.08 (13.04-82.17)	0.805
Tetrahydrocortisone	2945.23 ± 1227.57	2475.24 (2048.97-3508.52)	3553.55 ± 2069.90	3309.55 (1849.42-4618.60)	0.457
Tetrahydro-11-dehydrocorticosterone	150.24 ± 105.70	128.47 (86.49-155.73)	225.46 ± 120.38	219.98 (147.50-285.70)	0.021
Tetrahydrocorticosterone	111.74 ± 78.87	88.60 (55.55-145.35)	263.49 ± 308.70	171.15 (130.89-218.91)	0.007
5α-tetrahydrocorticosterone	506.93 ± 365.63	372.36 (248.61-644.59)	523.05 ± 322.54	422.25 (322.42-679.77)	0.570
Tetrahydrocortisol	1023.23 ± 509.55	806.60 (644.98-1413.56)	1259.60 ± 732.54	1090.40 (783.90-1613.18)	0.389
5α-tetrahydrocortisol	1890.18 ± 974.22	1667.86 (1081.87-2481.29)	1828.22 ± 1342.45	1169.83 (910.61-2463.39)	0.550
α-cortolone	1385.42 ± 589.98	1418.05 (1031.91-1485.55)	1939.85 ± 958.08	1920.14 (1136.76-2691.38)	0.108
ß-cortolone	455.92 ± 162.60	426.26 (306.91-544.40)	562.83 ± 298.18	517.45 (329.28-698.16)	0.389
α-cortol	295.59 ± 110.13	267.53 (197.42-385.50)	366.15 ± 202.33	326.41 (198.40-485.90)	0.439
ß-cortol	396.95 ± 158.67	346.97 (284.75-519.40)	507.68 ± 247.69	444.34 (304.95-690.83)	0.260
Cortisone	161.39 ± 68.35	160.62 (94.94-204.73)	138.02 ± 76.14	127.17 (86.74-159.49)	0.182
Cortisol	171.26 ± 80.21	144.39 (111.04-236.02)	150.62 ± 82.92	133.94 (95.13-169.10)	0.439
20α-dihydrocortisone	31.40 ± 8.99	32.56 (25.03-35.42)	20.83 ± 9.41	18.64 (13.57-26.51)	<0.001
20ß-dihydrocortisone	81.37 ± 32.81	82.00 (56.88-94.91)	96.69 ± 46.91	87.13 (61.93-118.11)	0.273
20α-dihydrocortisol	45.34 ± 24.50	40.58 (29.55-56.03)	82.24 ± 59.02	73.33 (46.20-95.88)	0.056
20ß-dihydrocortisol	239.06 ± 215.31	153.58 (106.30-271.14)	116.42 ± 92.01	79.80 (70.89-138.73)	0.033
6ß-hydroxycortisol	49.62 ± 20.01	45.56 (34.14-66.90)	49.10 ± 39.96	37.56 (25.15-57.61)	0.405

Supplementary Table S3. List of steroid metabolites (μg/24h): differences between the normal weight and obesity in prepubertal girls

	Normal weight (n=6)		Obesity (n=18)		*p* value
	Mean+SD	Median (IQR)	Mean+SD	Median (IQR)	
Androsterone	174.85 ± 136.71	120.31 (93.88-207.23)	559.06 ± 805.96	254.92 (158.10-440.15)	0.056
Etiocholanolone	74.98 ± 36.52	88.00 (42.75-104.48)	338.83 ± 567.60	119.75 (77.84-428.66)	0.177
Dihydroandrosterone	11.18 ± 8.01	8.04 (5.12-17.91)	27.99 ± 41.32	13.35 (9.70-20.35)	0.224
Dehydroepiandrosterone	54.31 ± 83.46	16.80 (8.34-38.92)	60.04 ± 55.59	35.87 (14.89-92.08)	0.310
Androstenediol	3.60 ± 1.24	3.72 (2.81-4.53)	18.15 ± 14.28	13.10 (8.17-24.34)	<0.001
11-oxo-etiocholanolone	192.52 ± 101.31	172.92 (140.22-201.07)	374.70 ± 182.12	340.02 (204.99-562.07)	0.027
5α-dihydrotestosterone	4.94 ± 3.03	4.60 (2.58-7.88)	5.73 ± 5.65	3.62 (2.46-6.53)	0.820
17ß-estradiol	0.63 ± 0.48	0.55 (0.22-0.93)	0.69 ± 0.76	0.38 (0.13-0.92)	0.820
Testosterone	6.03 ± 1.09	6.60 (5.27-6.88)	10.27 ± 8.77	6.86 (4.76-12.80)	0.494
11ß-hydroxyandrosterone	237.88 ± 78.82	248.68 (193.35-297.23)	464.53 ± 354.67	294.97 (269.92-496.48)	0.119
11ß-hydroxyetiocholanolone	100.49 ± 67.34	80.30 (45.20-165.88)	156.59 ± 118.31	134.81 (69.14-183.44)	0.378
17-hydroxypregnanolone	4.89 ± 3.25	4.69 (2.03-7.69)	8.34 ± 5.85	6.20 (4.94-8.78)	0.310
16α-hydroxy-DHEA	26.02 ± 17.52	22.80 (10.26-37.18)	143.12 ± 167.63	87.83 (40.18-177.55)	0.007
Pregnanediol	45.19 ± 25.73	46.93 (24.93-56.67)	179.83 ± 315.11	59.23 (25.61-153.88)	0.310
Pregnanetriol	118.46 ± 47.75	122.10 (91.38-160.29)	231.50 ± 155.55	154.70 (120.76-288.10)	0.077
Androstenetriol	18.39 ± 14.85	12.18 (7.77-27.92)	87.73 ± 158.25	50.48 (21.96-72.81)	0.007
Tetrahydro-11-deoxycortisol	48.00 ± 21.60	41.19 (33.30-62.20)	62.94 ± 37.89	58.51 (41.77-80.33)	0.415
Tetrahydrodeoxycorticosterone	3.95 ± 2.09	2.98 (2.71-4.58)	5.05 ± 4.29	3.77 (2.41-5.96)	0.770
Estriol	0.46 ± 0.38	0.30 (0.19-0.58)	1.50 ± 3.21	0.66 (0.31-0.87)	0.280
Pregnanetriolone	5.06 ± 2.12	4.61 (3.60-6.10)	11.89 ± 6.40	11.31 (7.10-13.58)	0.004
Pregnenetriol	2.21 ± 2.25	0.99 (0.94-2.32)	5.23 ± 4.11	3.84 (2.04-7.92)	0.040
Tetrahydrocortisone	1400.46 ± 528.56	1095.99 (1004.73-1782.82)	2430.33 ± 1348.91	2127.69 (1533.62-3063.53)	0.119
Tetrahydro-11-dehydrocorticosterone	81.35 ± 38.35	62.86 (56.25-112.84)	152.03 ± 86.77	127.35 (92.02-226.42)	0.119
Tetrahydrocorticosterone	55.45 ± 27.72	51.86 (32.04-75.67)	102.93 ± 88.36	74.10 (46.23-144.00)	0.251
5α-tetrahydrocorticosterone	251.21 ± 148.21	171.19 (135.52-352.32)	320.94 ± 184.71	259.68 (193.91-411.47)	0.392
Tetrahydrocortisol	381.42 ± 135.12	375.43 (267.10-459.76)	728.96 ± 640.79	524.12 (314.00-842.95)	0.156
5α-tetrahydrocortisol	660.27 ± 208.89	718.48 (464.54-820.75)	1059.30 ± 524.39	943.37 (575.19-1456.55)	0.119
α-cortolone	606.22 ± 326.37	433.01 (346.67-894.30)	985.07 ± 433.43	819.46 (686.16-1345.50)	0.056
ß-cortolone	239.72 ± 77.17	222.09 (174.57-297.33)	355.89 ± 185.70	303.69 (243.18-409.55)	0.199
α-cortol	118.60 ± 47.19	106.16 (86.97-140.02)	161.03 ± 82.74	135.87 (104.74-235.40)	0.280
ß-cortol	173.06 ± 49.52	160.42 (154.92-206.40)	368.15 ± 312.78	181.64 (145.53-538.56)	0.224
Cortisone	76.06 ± 31.32	73.18 (46.14-106.03)	86.36 ± 50.47	70.90 (56.89-92.58)	0.820
Cortisol	85.94 ± 41.13	79.00 (56.38-107.89)	83.66 ± 43.94	65.58 (49.25-109.48)	0.820
20α-dihydrocortisone	13.37 ± 6.49	11.65 (7.56-18.35)	11.10 ± 5.41	10.45 (6.69-14.50)	0.626
20ß-dihydrocortisone	34.68 ± 19.03	30.47 (18.28-43.73)	48.40 ± 25.73	43.63 (30.16-65.14)	0.415
20α-dihydrocortisol	29.11 ± 21.20	23.31 (11.75-39.65)	43.80 ± 43.70	29.75 (16.12-52.89)	0.673
20ß-dihydrocortisol	180.24 ± 142.27	158.42 (97.97-195.69)	56.64 ± 26.48	53.77 (38.26-70.88)	0.033
6ß-hydroxycortisol	30.80 ± 12.02	31.11 (19.20-40.97)	33.86 ± 34.35	18.43 (14.44-33.95)	0.378

Supplementary Table S4. List of steroid metabolites (μg/24h): differences between the normal weight and obesity in girls with advanced puberty.

	Normal weight (n = 15)		Obesity (n = 40)		*p* value
	Mean+SD	Median (IQR)	Mean+SD	Median (IQR)	
Androsterone	1855.48 ± 1183.85	1831.69 (999.00-2100.43)	1970.30 ± 1732.32	1374.73 (1043.89-2252.31)	0.874
Etiocholanolone	1347.82 ± 1076.99	719.82 (558.32-1965.09)	1116.51 ± 795.40	940.08 (616.55-1221.95)	0.786
Dihydroandrosterone	35.37 ± 24.83	28.15 (20.82-43.78)	52.90 ± 46.06	39.02 (22.43-62.25)	0.204
Dehydroepiandrosterone	194.52 ± 273.92	36.55 (22.98-268.66)	485.75 ± 1272.88	64.94 (31.93-270.22)	0.248
Androstenediol	37.57 ± 19.99	31.58 (23.42-55.69)	112.84 ± 257.65	50.26 (24.67-86.16)	0.155
11-oxo-etiocholanolone	657.19 ± 578.88	461.27 (278.30-740.38)	432.58 ± 294.00	315.47 (211.39-657.45)	0.233
5α-dihydrotestosterone	17.23 ± 16.26	15.13 (7.41-20.31)	1699 ± 11.89	14.41 (6.70-23.66)	0.772
17ß-estradiol	1.98 ± 1.48	1.29 (0.99-2.12)	2.20 ± 1.51	1.78 (1.07-3.16)	0.594
Testosterone	22.38 ± 18.24	17.80 (11.27-26.78)	26.49 ± 30.85	17.12 (11.22-28.22)	0.948
11ß-hydroxyandrosterone	1340.76 ± 906.05	1230.72 (845.54-1539.83)	1227.75 ± 1153.84	878.21 (678.43-1366.89)	0.280
11ß-hydroxyetiocholanolone	437.43 ± 647.75	183.72 (66.22-532.20)	297.90 ± 271.35	213.27 (105.07-428.82)	0.758
17-hydroxypregnanolone	23.76 ± 18.58	17.77 (11.54-32.73)	31.31 ± 33.63	17.30 (11.98-36.15)	0.715
16α-hydroxy-DHEA	330.74 ± 278.34	239.16 (110.22-441.75)	492.77 ± 541.93	310.61 (129.42-655.75)	0.394
Pregnanediol	305.23 ± 242.07	283.18 (118.26-378.62)	410.96 ± 385.88	263.69 (118.27-590.21)	0.531
Pregnanetriol	646.79 ± 459.42	522.58 (309.92-839.83)	647.75 ± 448.90	536.43 (372.78-804.78)	0.859
Androstenetriol	278.36 ± 164.39	244.31 (169.67-365.42)	389.03 ± 394.39	264.62 (133.49-497.42)	0.715
Tetrahydro-11-deoxycortisol	74.57 ± 32.87	69.26 (46.15-97.60)	65.32 ± 49.56	50.48 (35.28-82.57)	0.161
Tetrahydrodeoxycorticosterone	7.77 ± 4.87	7.43 (3.33-11.68)	7.93 ± 6.88	4.76 (3.34-9.24)	0.801
Estriol	5.58 ± 4.14	4.62 (2.66-7.39)	9.87 ± 10.78	5.33 (2.85-12.97)	0.415
Pregnanetriolone	16.25 ± 12.20	12.58 (8.28-20.78)	14.64 ± 14.92	9.66 (6.53-14.23)	0.298
Pregnenetriol	32.69 ± 43.78	14.71 (6.28-31.15)	183.98 ± 400.41	33.68 (16.58-162.44)	0.045
Tetrahydrocortisone	3151.05 ± 1042.78	3278.60 (2244.49-3738.04)	3059.06 ± 2157.01	2502.48 (1758.73-3782.87)	0.325
Tetrahydro-11-dehydrocorticosterone	155.36 ± 90.49	120.51 (90.37-219.57)	152.30 ± 109.34	133.60 (69.73-184.73)	0.874
Tetrahydrocorticosterone	105.95 ± 56.94	100.34 (57.42-127.91)	145.57 ± 129.56	98.54 (72.33-153.44)	0.531
5α-tetrahydrocorticosterone	346.19 ± 111.17	328.53 (247.47-426.39)	384.70 ± 301.72	346.85 (176.26-462.38)	0.568
Tetrahydrocortisol	1132.53 ± 499.81	1101.57 (716.61-1388.38)	1428.86 ± 1372.95	1147.31 (735.09-1704.99)	0.660
5α-tetrahydrocortisol	1281.04 ± 489.55	1054.00 (937.81-1687.02)	1783.34 ± 1591.22	1353.33 (899.26-2039.96)	0.460
α-cortolone	1816.33 ± 944.00	1551.04 (1171.45-2324.15)	1512.81 ± 904.17	1281.58 (886.97-1972.49)	0.226
ß-cortolone	619.88 ± 263.36	530.65 (417.21-803.06)	471.40 ± 282.66	440.44 (277.20-563.86)	0.041
α-cortol	306.05 ± 155.54	287.77 (197.85-376.05)	407.72 ± 452.84	288.02 (178.13-445.30)	0.786
ß-cortol	515.55 ± 293.11	399.39 (289.54-703.57)	482.70 ± 396.98	391.37 (269.72-508.36)	0.518
Cortisone	134.79 ± 38.84	122.85 (106.24-174.40)	123.04 ± 76.05	106.63 (68.17-157.36)	0.134
Cortisol	150.55 ± 51.22	138.12 (110.07-165.87)	143.19 ± 95.49	127.30 (78.18-167.21)	0.233
20α-dihydrocortisone	22.23 ± 8.01	21.76 (14.04-28.83)	17.46 ± 12.23	13.65 (10.54-23.89)	0.014
20ß-dihydrocortisone	70.59 ± 31.13	64.92 (41.76-101.77)	83.25 ± 68.08	62.65 (46.17-102.37)	0.903
20α-dihydrocortisol	52.58 ± 35.57	38.46 (29.04-62.95)	88.59 ± 154.17	40.41 (25.39-62.72)	0.903
20ß-dihydrocortisol	252.33 ± 209.11	223.02 (111.69-300.32)	110.03 ± 81.43	82.34 (61.37-119.73)	0.004
6ß-hydroxycortisol	50.17 ± 27.93	40.35 (28.46-76.89)	44.40 ± 38.78	34.40 (20.68-49.01)	0.344

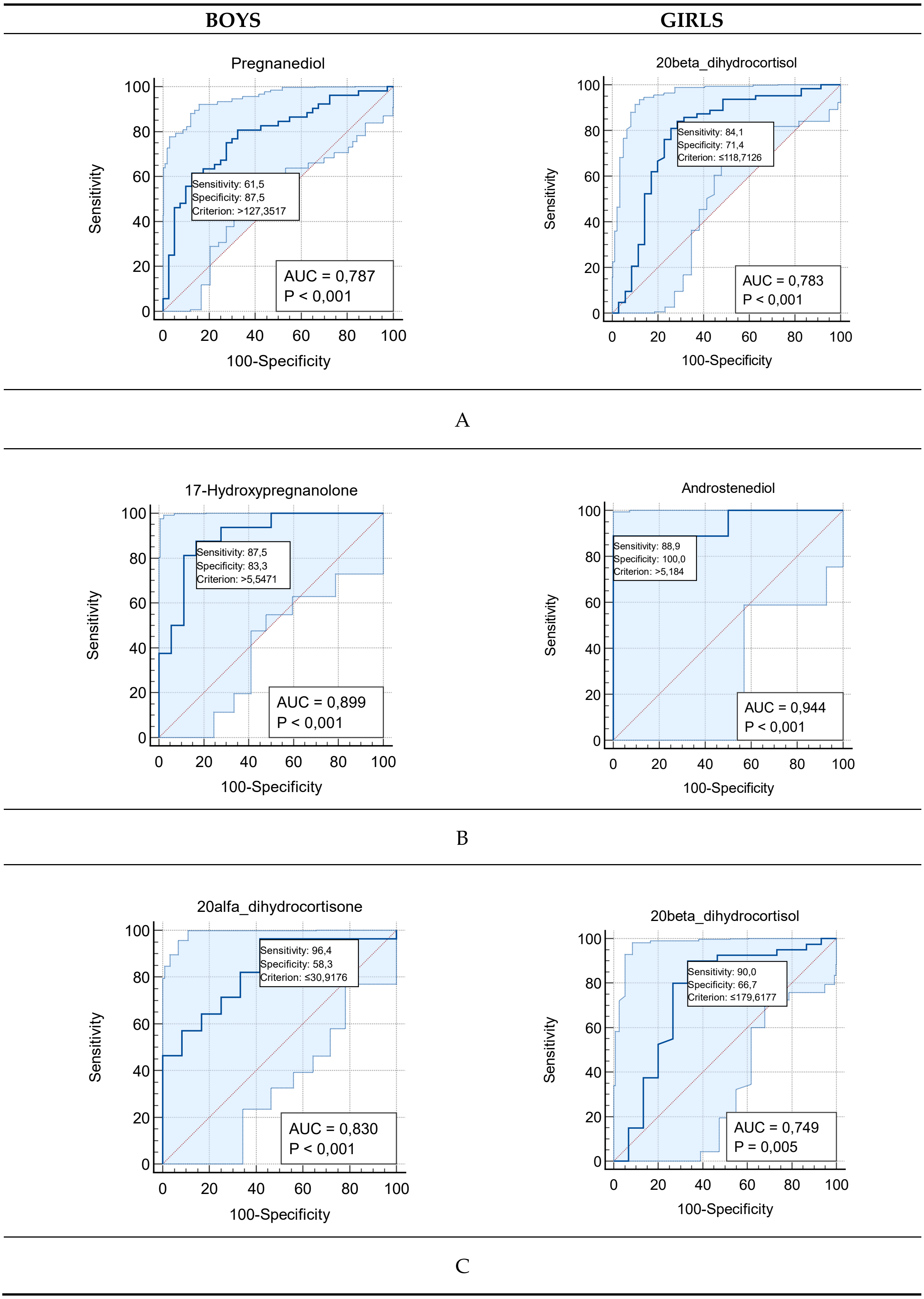

Supplementary Figure S1. Results of the analysis of the ROC curve for most appropriate classifier to distinguish alterations in steroid metabolites between normal weight and obesity. The blue shaped area is the 95% confidence interval of the sensitivity at the given specificity. A – whole group divided by sex, B – prepubertal group divided by sex, C – group with advanced puberty divided by sex.
